# Sympathetic nerve blocks for posttraumatic stress disorder: an evidentiary review for future clinical trials

**DOI:** 10.3389/fpsyt.2023.1309986

**Published:** 2023-12-22

**Authors:** Sakshi Prasad, Nityanand Jain, Tungki Pratama Umar, Igor Radenkov, Sirwan Khalid Ahmed, Virginia Sakagianni, Sofia Kollia, Mohmed Junaid Hingora, Nikita Kumari, Amir Reza Akbari, Lubova Renemane, Anil Bachu

**Affiliations:** ^1^Faculty of Medicine, National Pirogov Memorial Medical University, Vinnytsia, Ukraine; ^2^Faculty of Medicine, Riga Stradinš University, Riga, Latvia; ^3^UCL Centre for Nanotechnology and Regenerative Medicine, Division of Surgery and Interventional Science, University College London, London, United Kingdom; ^4^Faculty of Medicine, St. Cyril and Methodius University, Skopje, North Macedonia; ^5^Department of Adult Nursing, College of Nursing, University of Raparin, Rania, Sulaymaniyah, Kurdistan, Iraq; ^6^School of Medicine, Aristotle University of Thessaloniki, Thessaloniki, Greece; ^7^School of Medicine, National and Kapodistrian University of Athens, Athens, Greece; ^8^Department of Medicine, Pandit Deendayal Upadhyay Medical, Rajkot, India; ^9^Sindh Medical College (SMC), Jinnah Sindh Medical University (JSMU), Karachi, Pakistan; ^10^Emergency Department, Nottingham University Hospitals NHS Trust, Queen's Medical Centre, Nottingham, United Kingdom; ^11^Department of Psychiatry and Narcology, Riga Stradinš University, Riga, Latvia; ^12^Baptist Health– UAMS Psychiatry Residency Education Program, North Little Rock, AR, United States

**Keywords:** stellate ganglion block, PTSD, ropivacaine, sympathetic nerve block, cervical sympathetic block, dual sympathetic block

## Abstract

Posttraumatic stress disorder (PTSD) is a chronic disorder resulting from exposure to traumatic events. In recent years, sympathetic nerve blocks have gained interest as an emerging treatment modality for PTSD. They have been shown to reduce autonomic dysfunction associated with PTSD symptoms, particularly in refractory and treatment-resistant patients. However, there is limited evidence regarding the technique’s effectiveness in PTSD patients. Therefore, this scoping review was designed to update and summarize the current literature on this topic to inform the design of future clinical trials and studies. Our review of 22 studies (mostly case reports and series) included 1,293 PTSD patients who received sympathetic nerve blocks, primarily military service members and veterans, with a median age of 42.2 years. 0.5% Ropivacaine was the preferred anesthetic, and the right sided stellate ganglion block was the most commonly used technique. Relapse of symptoms was reported commonly, resulting in additional nerve block sessions. Most reported side effects were mild and transient. Despite the encouraging results, we remain cautious in interpreting the benefit of the technique due to the lack of sufficient standardized clinical trial data, heterogeneity in reported results, and the potential for bias in reporting. Future studies should focus on evaluating and addressing the technique’s effectiveness, safety, tolerability, and indications.

## 1 Introduction

Posttraumatic stress disorder (PTSD) is a chronic disorder that occurs after exposure to actual or imminent serious injury, sexual violence, or death by either direct or indirect experience including repeated exposure to the details of the traumatic event at workplace (1). Because PTSD often affects people exposed to natural catastrophes, military combat, physical or sexual abuse, or a near-fatal crash, it has been classified as a trauma-related disorder to distinguish it from anxiety and depression (2). Characteristic symptoms include re-experiencing traumatic events, avoidance of trauma triggers, and increased arousal, such as sleep disturbance, irritability, and a persisting sense of increased current threat. Although many of these symptoms overlap with acute stress disorders, symptoms in patients with PTSD typically tend to last longer than one month (3, 4). While exposure to traumatic events is the primary triggering event in PTSD, the exact pathogenesis of PTSD is not well understood. Biological and psychosocial risk factors are being increasingly recognized as predictive factors for disease clinical course and symptom severity (5).

Trauma-focused cognitive-behavioral therapy (CBT) is universally recommended as a first-line psychological treatment for PTSD, according to a review of 14 international PTSD treatment guidelines (6). Eye Movement Desensitization and Reprocessing (EMDR) has also been a commonly recommended alternative to CBT as a first-line psychological therapy (6, 7). These techniques may be applied alone or in combination with pharmacotherapeutic agents like selective serotonin reuptake inhibitors, serotonin-noradrenaline reuptake inhibitors, and tricyclic antidepressants. Concerns about difficulties in integrating discussion of trauma, organizational and structural barriers, and the availability of safe spaces persist despite compelling clinical evidence of the effectiveness of behavioral therapy techniques (8). Because these techniques rely on re-experiencing traumatic events, patient preferences, recall, and focus along with clinician expertise have a profound impact on treatment effectiveness.

Accordingly, there has been an increase in interest in alternative approaches to PTSD treatment. One such technique is to use neural sympathetic blocks, specifically the stellate ganglion block (SGB). The stellate or cervicothoracic ganglion, found in approximately 80% of the population, is formed by the fusion of the inferior cervical and first thoracic ganglia (9). In this outpatient procedure, a local anesthetic is injected into the stellate ganglion, blocking sympathetic outflow (10). Since most PTSD symptoms are believed to arise from an altered autonomic nervous system (11), the blockage procedure attenuates autonomic dysfunction, directly targeting the source of the PTSD stimulus (12, 13). When used as an alternative or concomitant treatment, it appears to be very effective in reducing symptoms associated with PTSD such as irritability, difficulty relaxing, difficulty concentrating, and difficulty sleeping by reducing sympathetic hyperactivity.

In recent years however, a dual sympathetic block (DSB), also called as cervical sympathetic block (CSB), has been described in the literature. In this technique, an additional nerve block is given at the level of C4 one week after the usual stellate block has been administered at the level of C6. It has been proposed that this additional block at the level of C4 is beneficial in prolonging the effects and improving the efficacy of SGBs. This prolonged effect is achieved by targeting an additional ganglion in the cervical sympathetic chain (14, 15). While Lipov et al., have suggested targeting the superior cervical ganglion, Mulvaney et al., have suggested targeting the middle cervical ganglion. Anatomically, middle cervical ganglion is the smallest of the cervical sympathetic chain ganglia and is often absent, but when present it is located at the level of C5-C6 (16). Superior cervical ganglion, on the other hand, is the largest and primary component of the cervical sympathetic chain located at C2-C3 level. However, anatomic variations are commonly noted in the localization of these ganglia, thereby making it difficult to conclude specifically which additional ganglion is targeted by the DSB technique. The rationale behind targeting a second ganglion lies in the path of the sympathetic fibers originating from these plexuses. While fibers from stellate ganglion follow the vertebral artery, the fibers from superior cervical ganglion follow the internal carotid artery, thereby innervating different regions of the brain (14).

Previous systematic reviews investigating the effectiveness of SGB for PTSD management have consistently found and reported favorable results. However, all reviews have reported the limitations in the study designs reported, and the limited number of individuals included in the reported studies. The reviews also have highlighted the overwhelming representation of war veterans in the studies, limiting the generalizability of the findings to non-military patients (17, 18). Additionally, none of the previous reviews considered the use of DSB/CSB for management of PTSD. Given these findings, the current study aimed to update and summarize the current knowledge on the use of sympathetic nerve blocks (SGB and DSB/CSB) for PTSD management. The rationale behind the review is to provide a background for basing future clinical trials and prospective studies investigating the role of sympathetic nerve blocks for PTSD management.

## 2 Methods

An initial scoping search of the current literature was conducted to ensure that no similar systematic review had been conducted in the last five years. A search of International Prospective Register of Systematic Reviews (PROSPERO) was also done to identify potentially similar study designs. This study protocol complied with the Preferred Reporting Items for Systematic Review and Meta-analyses (PRISMA) 2020 statement. However, the study protocol was not registered with PROSPERO since the present study was a scoping review.

### 2.1 Data sources and search strategy

A search of six medico-scientific databases was conducted – PubMed, PubMed Central, Scopus, Web of Science, Cochrane Library, and ClinicalTrials.gov. All databases were searched utilizing a comprehensive search strategy comprising of both MeSH terms and free text to ensure all relevant studies were identified. The following search terms were used for literature search - (“stellate ganglion block” OR “SGB” OR “stellate ganglion nerve block” OR “cervicothoracic ganglion block” OR “cervicothoracic sympathetic block” OR “stellate ganglion nerve block” OR “sympathetic block” OR “cervical sympathetic block” OR “CSB” OR “dual sympathetic block” OR “DSB” OR “autonomic nerve block” OR “sympathetic blockade”) AND (“PTSD” OR “post-traumatic stress disorder” OR “posttraumatic stress disorder” OR “delayed-stress disorder” OR “delayed-stress syndrome” OR “post-traumatic stress syndrome” OR “trauma-related disorder”). To identify potential studies that met the eligibility criteria, the reference lists of included and cited articles were manually screened.

### 2.2 Eligibility criteria

We included studies that reported on the administration of SGB to patients with PTSD and were published between January 2002 and October 2023. We included interventional original articles, randomized control trials, case series, and case reports that reported on adult human patients. However, review articles and studies that addressed more than one disorder other than PTSD, pediatric populations, incomplete registry studies, posters, editorials, letters, and animal studies were excluded from this review.

### 2.3 Screening process

At the end of the database search, all the relevant citations were transferred to an Excel spreadsheet. An individual reviewer (I.R., S.K.A., V.S., S.K., M.J.H., N.K.) was assigned a single database for screening and data extraction. To assess the eligibility of the retrieved studies (based on the inclusion and exclusion criteria), the reviewer independently screened the titles and abstracts of the retrieved studies. In the second step, the results of screening and data extraction were verified by two other reviewers not involved in the screening and extraction process (T.P.U. and A.R.A.). The disagreement rate varied from 1 to 4% based on the database. Disagreements were sorted using a collaborative approach which included the initial reviewer of the database, the two validators, and two other independent reviewers not involved in screening, extraction, or validation process (S.P. and N.J.). Next, full texts of the included studies were analyzed and verified.

### 2.4 Risk of bias assessment

The Risk of Bias (RoB) assessment was performed using the relevant JBI critical appraisal tool list based on different study designs included in the review. For the randomized controlled trials and non-randomized controlled trials, we used the ROB-2 and ROBINS-I tool, respectively. To assess the overall quality of the studies, a cumulative perceived risk of bias category was assigned based on the checklist results, categorizing them as low risk, moderate risk, or high risk. RoB assessment followed the same workflow methodology as mentioned above for the screening process.

### 2.5 Data extraction

Independent reviewers extracted the following data from the included studies: (i) study design; (ii) study blinding type; (iii) country of study, (iv) sample size, (v) socio-demographic data of the participants including the type of trauma witnessed/experienced by the participants; (vi) use of concomitant therapy; (vii) laterality of SGB; (viii) anesthetic used; (ix) technique procedure; (x) number of sessions; (xi) outcome measures; (xii) follow-up period; (xiii) adverse effects; (xiv) dropout rates; and (xv) reported limitations.

## 3 Results

**Figure 1:**
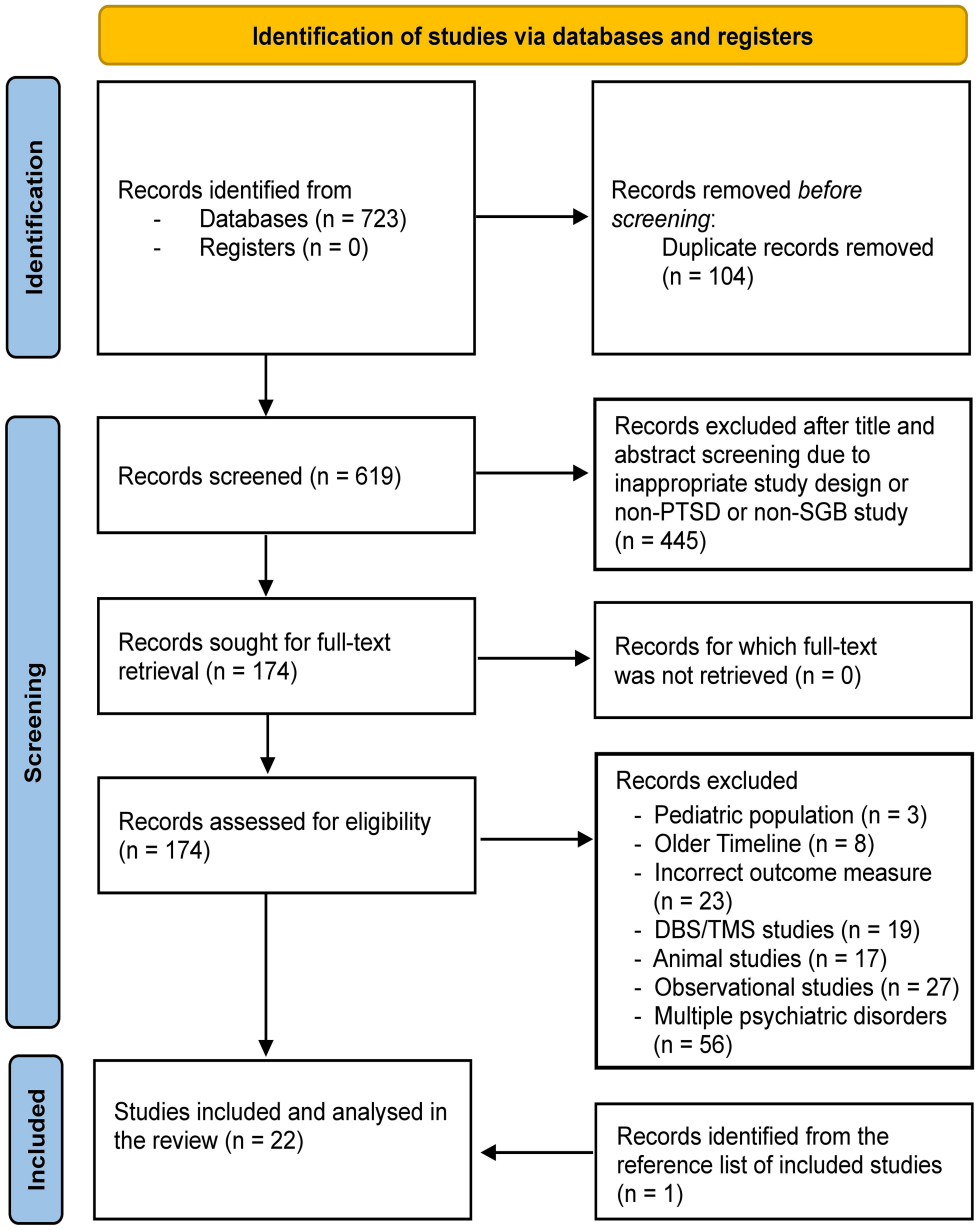
The PRISMA flowchart for the present study. DBS, deep brain stimulation; TMS, transcranial magnetic stimulation; PTSD, posttraumatic stress disorder; SGB, stellate ganglion block.

Our search yielded a total of 723 records across all databases reviewed (Table 1). The PRISMA flowchart (Figure 1) shows that after removing duplicates, 619 unique records were identified for title and abstract screening. Four hundred and forty-five records were removed at this stage. Among the remaining 174 studies, 153 were removed due to various reasons (Figure 1). Studies published prior to the early 2000s were removed due to non-clinical introduction of commonly used anesthetic agents like ropivacaine. Some studies were also removed due to incorrect outcome measure, i.e., the studies did not use specific PTSD symptom scales like PCL-5. One study was added after screening the reference lists of the included studies, leading to a total of 22 studies investigated in the present review.

**Table 1:** Search Results from individual databases.

Database	Search results	Database	Search results
PubMed	41	Web of Science	79
PubMed Central	225	Cochrane Library	21
Scopus	348	ClinicalTrials.gov	9
Total			723

All of the included studies were reported from the United States (US). Based on the study design, there were seven case series (19–25), six case reports (26–31), six retrospective cohort studies (14, 15, 32–35), two randomized controlled trials (36, 37), and one non-randomized clinical trial (38). The risk of bias assessment is presented in Supplementary file S1.

### 3.1 Patient characteristics

The reviewed studies included a total of 1,347 participants, with 1,293 (96%) participants receiving SGB and 54 (4%) participants allocated to the placebo (sham) group. Among the participants who received SGB treatment, the median age was 42.2 years (range 17–81 years). Among the patients receiving SGB, there were 560 male (43%) and 354 female (27%) participants. The sex for remaining participants was not reported and could not be ascertained from the text (30%). A summary of study outcomes is provided in Table 2.

**Table 2:** Overview of study design, clinical characteristics and outcomes reported in the included studies.

Study	Study design	Sample size	Complaints/history	Concomitant pharmacotherapy	Concomitant psychotherapy	Measurement instruments	PCL-M baseline	PCL-M after block	Main outcomes	Dropout rates	Limitations
Lipov et al. (28)	Single group (UB)	1 male patient	Severe anxiety, sporadic nausea, shaking, loss of appetite, and insomnia. Patient began smoking after 10 years of cessation	Escitalopram, alprazolam, and olanzapine	Relaxation training (recorded tape)	None reported. PTSD diagnosis was guided by the clinical evaluation done by a neuropsychologist with expertise in PTSD	None	None	One-week post-SGB the anxiety level was reduced by 80 to 90%, appetite had improved approximately 50%, and sleep was about 25% better. 90% improvement after 3 months (self-reported)	None	None reported
Lipov et al. (31)									Self-reported functioning at 70% of the pre-trauma level at 5 months after SGB		
Mulvaney et al. (25)	Single group (UB)	2 male patients	Insomnia, nightmares, heart palpitations, and shortness of breath. One patient had drug-induced erectile dysfunction	Sertraline, quetiapine, trazadone, venlafaxine, and zolpidem	None	PCL	54.00–76.00	24.00–34.00	Immediate, significant, and durable relief as measured by the PCL (minimum 17, maximum 85). Both patients discontinued antidepressant and antipsychotic medications while maintaining their improved PCL score. Erectile dysfunction went away due to tapering of medications	None	Further investigation of SGB use is warranted
Hicky et al. (20)	Single group (UB)	9 patients	Patients were undergoing PTSD treatment before enrolment in the study	SSRI	Individual cognitive processing therapy or PE therapy	CAPS	None	None	Five of the nine patients (56%) experienced a clinically significant (>30%) reduction in PTSD symptoms	None	Need clarification from RCT
Lipov et al. (32)	Single group (UB)	7 male and 1 female patient	6 patients were taking three or more different medications to treat PTSD or related symptoms like depression (unspecified)	Not reported	Not reported	PCL-M	55.00–79.00	21.00–63.00	75% of the patients experienced a substantial decline in PTSD symptom severity (range 47–73%) The remaining 25% of the patients benefited from a decrease in symptoms, but the results were not clinically meaningful (6 and 12.1%) Patients who underwent two or more SGB sessions experienced greater levels of PTSD symptom relief (decreases in severity score of 58.6 and 73.4%)	None	None reported
Lipov et al. (29)	Single group (UB)	1 male patient	Nightmares, night sweats, daytime flashbacks, heart palpitations, self-isolation, mood swings, insomnia, poor memory. Patient had alcohol dependence	Citalopram	Group and individual psychotherapy	Neuropsychologic testing, PCL-M	71.00	30.00	Reduction of PCL-M by 57.7% by the end of second SGB, in addition to an increase in immediate memory (50%), recent memory (58%), and recognition memory (36%). Patient stopped drinking alcohol except at social events	None	Need more epidemiologic evidence
Alino (19)	Single group (UB)	4 male patients	Nightmares, insomnia, hypervigilance. Two patients had alcohol dependence, two attempted suicides	Citalopram, buspirone, fluoxetine, bupropion, prazosin, and sertraline	Individual psychotherapy. One patient underwent CBT and PE therapy	PCL-M, PCL-C	64.00–85.00	18.00–34.00	Right-sided SGB (RSGB) at C6 has an excellent safety profile that may provide sustained relief of PTSD symptoms	25.00%	Not RCT
Mulvaney et al. (24)	Single group (UB)	166 patients	Not reported. The majority of patients had at least once suffered from mild to moderate head trauma. 2 patients reported concussions and 2 patients reported traumatic brain injury	Five patients were taking a single psychotropic medicine. 12 started SSRI during follow-up period after SGB	One patient underwent eye movement desensitization and reprocessing	PCL-M; interview with a clinical psychologist or a senior military physician	50.30	32.50	After 1 week, 78.6% of patients improved with a reduction of 10 or more (mean 22) in PCL score. After 1/2 months 81.76% had a mean reduction of 22 in PCL score After 3 to 6 months 73.5% had a mean reduction of 21.8 in PCL score	54.80%	Incomplete data collection, especially on second blocks. Limited ability to draw stronger conclusions on the potential efficacy of second blocks
Mulvaney et al. (23)	Single group (UB)	10 male and 1 female patient	Not reported	Not receiving any therapy at least 6 months prior to SGB		PCL-M; Joggle Research automated battery of psychometric measures; psychomotor vigilance test	61.00	31.91	Significant improvement in mean response was observed for VOLT (mean change: 95.6; [95% CI, 35.15–155.94]; *p* = 0.005), NBACK (mean change: 189.1; [95% CI, 84.52–293.66]; *p* = 0.002), PVT (mean change: 151.6; [95% CI, 25.48–277.79]; *p* = 0.023), MPT (mean change: 9.64; [95% CI, 3.09–16.18]; *p* = 0.008), and PCL-M (mean change: −29.18; [95% CI, −34.09 to −24.27]; *p* < 0.001)	None	Further research is necessary to evaluate these effects for longer periods of time
Lynch et al. (33)	Single group (UB)	30 male patients	The patients represented the first 30 patients enrolled in the larger study by (24)	3 patients received pharmacotherapy for PTSD	The same 3 patients also were enrolled in psychotherapy sessions	PCL-M; interview with a clinical psychologist or a senior military physician	48.69	32.15	1 week after the procedure, 16 points change in PCL-M mean score (48.69 to 32.15) was noted. 2–4 months after the procedure, subjects continued to show improved PTSD symptoms (PCL-M = 31.88)	13.33%	None reported
Hanling et al. (36)	Crossover assignment (DB)	23 male and 4 female patients; 15 patients randomized to placebo group	Not reported	20 patients received unspecified mental health treatment		Mini International Neuropsychological Interview; CAPS; PHQ-9, Beck Anxiety Inventory, Sheehan Disability Scale, and Visual Analog Scale for pain	66.85 (SGB), 64.20 (sham)	58.92 (SGB), 57.25 (sham)	PTSD, anxiety, and depression scores all showed improvement across time, but there was not statistically or clinically relevant difference in outcomes between the active and control groups	None	No RCT as a base of power analysis Skewed sample sizes Using a 2:1 randomization and unidirectional crossover from the sham group to the active SGB group was less than ideal
Rae Olmsted et al. (37)	Two-arm parallel assignment (SB)	64 male and 10 female patients; 39 patients randomized to placebo group	Depression, anxiety, diminished physical functioning, pain	Patients reported use of antidepressants (41%), anxiolytics (18%), opioids (3%) and other drugs (26%) like antihypertensives, anticonvulsants	38 (51%) of SGB group received concomitant psychotherapy	PCL-C; CAPS-5 TSSS; PCL-5; PHQ-9; GAD-7; K-6 Distress scale; SF-12 survey	41.54 (SGB), 43.23 (sham)	29.49 (SGB), 38.11 (sham)	Significant improvement of CAPS-5 TSSS (mean change: −12.16), PCL-5 (mean change: −12.63), PHQ-9 (mean change: −4.11), GAD-7 (mean change: −4.42), K-6 Distress Scale (mean change: −2.52), pain (mean change: −0.56), SF-12 mental (mean change: 1.74), and SF-12 physical (mean change: 2.56)	6.76%	Treating physicians were unable to be blinded Participants’ potential recognition of signs of Horner syndrome Limited clinical generalizability
Mulvaney et al. (15)	Two groups (UB)	62 male and 41 female patients received right SGB; 34 males and 10 females received left CSB	Not reported	Not reported	Not reported	PCL-5	Average 60.90 for right SGB; average 58.50 for left CSB	Average 35.70 for right SGB; average 26.72 for left CSB	Single SGB and CSB are both effective in treating PTSD. A CSB is safe and may be more effective than a standard SGB in the treatment of PTSD.	50.00% in the right SGB group; 0.00% in the left CSB group	Patients lost-to follow-up and limited follow-up duration. Retrospective study design
Odosso et al. (35)	Single group (UB)	35 patients	Not reported	Not reported	Procedural Education program	PCL-M; pre- and post-procedure semi-structured interviews	49.30	36.10	Although a consistent 23 to 25% reduction in reported symptoms was noted, the treatment plateaued at that level A regression toward baseline PCL-M was noted Stigma associated with mental health treatment was significantly less with the SGB Service members were more likely to engage in other forms of therapy when the procedure was at its maximum effect	26.00%	Highly individualized response associated with the SGB It was impossible to predict, control, or remove all individual personal triggers The findings may not generalize to other units or other deployments
Lipov et al. (14)	Single group (UB)	178 male and 138 female patients	62 patients had attempted suicide (29 males and 33 females)	Not reported	Not reported	PCL-M, PCL-C	Above 35.00	Not reported	In male patients, the change in PCL scores before and after intervention were significantly different based on background of the patient (military vs. non-military change – 32 vs. 25 points; *p* = 0.012) In female patients similar results were observed (military vs. non-military change – 39 vs. 28 points; *p* = 0.020) In both genders, there was no significant difference in PCL scores based on age groups, suicide history, or use of psychiatric medications PCL scores from pre- to post-intervention for all participants showed a drop greater than 10 points in 81% of patients, greater than 15 points in 74.9% of patients, and greater than 20 points in 69.7% of patients	None	Inconsistent patient follow-up, retrospective study design, and single physician administration
Mulvaney et al. (34)	Single group (UB)	4 male and 6 female patients received right sided SGB followed by left sided SGB; 195 patients received right sided SGB only	10 patients receiving both right- and left-sided SGB were nicotine smokers	Not reported	Not reported	PCL-5	55.10	26.80	117 patients out of 205 showed improvements after right SGB. 20 patients were refractory to right SGB. 10 of these 20 patients reported that PCL-5 score 1 week post left-SGB dropped on average by 28.3 points compared to 6.2 points post right-SGB. In these 10 patients, this difference between left and right SGB was statistically significant (*p* = 0.010).	9.09%	Limited access to treatment for the patients may introduce sampling bias Small sample size, retrospective design, and short-term follow-up make it difficult to form generalizable conclusions The possibility of an additive effect
Peterson et al. (38)	Single group (UB)	8 male and 3 female patients	Not reported	Not reported	10 massed PE sessions of 90 min each over 2 consecutive weeks	CAPS-5; PCL-5; PHQ-9	53.42	21.41	Participants reported a mean post-treatment PTSD symptom reduction of 32 points on the PCL-5. Most participants (90.9%) demonstrated clinically significant change on the PCL-5 (i.e., ≥10 points) by the final treatment session and 50.0% no longer met the diagnostic criteria for PTSD at 1-month follow-up	16.67%	It is difficult to extrapolate the degree to which this rapid symptom reduction can be attributed to massed PE, SGB, or the combination of the two modalities
Block et al. (26)	Single group (UB)	1 male patient	Depression, anxiety, panic attacks, mood swings, avoidance behavior, insomnia	Not reported	Not reported	PCL-5	73.00	20.00	Use of pulsed radiofrequency (PRF) along with bilateral SGB improves duration of symptom relief	None	Single patient experience
Lipov et al. (30)	Single group (UB)	1 male patient	Alcohol and narcotics dependency, bipolar disorder, impulsivity and disordered sleep	None	None	BDI, BAI, PCL-5, CHRT	42.00	11.00	There was 93.5% reduction in depression scores, 86.7% reduction in anxiety scores, and 73.8% reduction in PTSD symptoms	None	None reported
Lipov and Faber (21)	Single group (UB)	2 male and 2 female patients	Rage, paranoia, night terrors, insomnia, hypertension, weight gain, depression, anxiety, suicide attempts	Not reported	Not reported	PCL-5	40.00–44.00	7.00–17.00	CSB is beneficial for treatment of non-responders and patients refractory to medications in comparison with single-level SGB	None	Differences in patient follow-up time points and uncontrolled case study design
Lynch et al. (22)	Single group (UB)	128 male and 137 female patients	Not reported	Not reported	Not reported	GAD-7, PCL-5	Not reported	Not reported	GAD-7 scores for the cohort decreased from 15.9 to 6.9 within 1 week of SGB, with 80% of the patients demonstrating clinically meaningful results GAD-7 scores after 1 month increased marginally to 7.6 with 76% patients demonstrating clinically meaningful results	None	None reported
Kuo and Nicklay (27)	Single group (UB)	1 female patient	Depression, anxiety, insomnia, irritability, hypervigilance, hyperhidrosis, chronic pain, muscle tension, seizures	SSRI, quetiapine	Talk therapy	PCL-5	57.00	2.00	Patient’s PCL-5 score dropped from 57.00 to 14.00 following two sessions of bilateral SGB 1 week apart Six weeks later the score dropped further 8.00. However, 2 weeks later the score increased back to 14.00 points At this time, she received a bilateral botox-enhanced SGB which reduced the score to 2.00	None	None reported

PTSD, posttraumatic stress disorder; BDI, beck depression inventory; BAI, beck anxiety inventory; CSB, cervical sympathetic blockade; CAPS-5 TSSS, clinician-administered PTSD scale for the Diagnostic and Statistical Manual of Mental Disorders, Fifth Edition total symptom severity scores; CHRT, concise health risk tracking self-report; CI, confidence interval; GAD-7, general anxiety disorder-7; K-6 Distress scale, Kessler psychological distress scale; MPT, motor praxis test; NBACK, n-back task cognitive test; PHQ-9, patient health questionnaire-9; PVT, psychomotor vigilance task; RCT, randomized controlled trial; SF-12, Short Form 12 Health Survey; SGB, stellate ganglion block; VOLT, visual object learning test; UB-unblinded or open label; SB, signal blind; DB-double blind; PCL-M, Posttraumatic Stress Disorder Checklist - Military Version (wherever applicable, an average value or range for the score has been provided if the study reported more than one patient); PCL- C, Posttraumatic Stress Disorder Checklist - Civilian Version; PCL-5, PTSD Checklist for DSM-5, CBT, cognitive behavioral therapy; PE, prolonged exposure, SSRIs, Selective serotonin reuptake inhibitors.

### 3.2 Trauma types among PTSD patients

Majority participants were active military personnel or retired military veterans who were involved in or witnessed presence in conflict zones or terrorist attacks. Eleven patients were first responders (firefighters, police) along with one patient who suffered from a motor vehicle accident (34). In another study, the authors identified 17 different types of traumas among 249 patients including divorce/family issues, domestic violence, sexual assault, childhood emotional abuse, head trauma, car accident, bullying/hazing etc. (14). From different studies, we also identified 12 individuals who reported childhood abuse and trauma including loss of loved one in car accidents (21, 26, 27). In another case series, the authors identified a couple with PTSD due to wrongful incarceration of the husband. The wife could not handle the nightmares and severe startle responses leading her to develop secondary PTSD (21).

Furthermore, one participant was reported to be a victim of a life-threatening assault during an armed robbery (28). The same patient’s six-month follow-up was, however, described in another publication by the same research team (31). For the purposes of this study, we counted this patient only once but considered both publications separate (to maintain alignment with indexation records). Similarly, in a follow-up study by Lynch et al. (33) 30 male participants who initially participated in a study by Mulvaney et al. (24) were counted only once, though both studies were recorded separately. In other cases, type of trauma was not identified in the text.

### 3.3 Choice of anesthetic agent

0.5% solution of Ropivacaine, a long-acting amide local anesthetic, was the choice of anesthetic agent in 11 studies. The dosage varied from 6 to 10 mL with 7 mL being the most frequently used dosage. Nine studies, from two research teams, reported the use of 7 to 8 mL 0.5% solution of Bupivacaine, another amide group local anesthetic (14, 21, 26–32, 31). When Bupivacaine was administered, the authors chose to supplement it with 5 μg/mL clonidine. Two studies did not report the anesthetic agent used for SGB.

### 3.4 Laterality of SGB

Fourteen studies reported the use of the right sided SGB technique, making it the most commonly performed SGB. The technique was administered to 700 study participants (54%), though its success varied. Ten patients were administered a left sided SGB after the right sided SGB failed to provide symptomatic relief (34). Three other studies did not report the laterality of SGB used.

### 3.5 Dual/cervical sympathetic block

Seven studies reported the use of dual sympathetic block (DSB) or cervical sympathetic block (CSB). In all studies, DSB was given bilaterally, except for two studies in which DSB was given unilaterally. 35% of the total participants received DSB of which 244 patients received right-sided DSB (14) while 44 patients received left-sided DSB (15). The remaining 168 patients received bilateral DSB (21, 22, 26, 27, 30).

### 3.6 SGB vs. unilateral DSB in PTSD

A single study compared the effectiveness of SGB with that of DSB. In the study, there were 44 patients who underwent left side DSB and 103 patients who underwent right side SGB (15). At the one-month follow-up timepoint, the authors noted that there were no significant differences in the PCL-5 score reduction between the two groups when compared with the baseline scores (mean reduction of 31.78 points in DSB group vs. 25.20 points in SGB group; *p* = 0.54). Despite the non-significant results, the higher mean reduction in DSB group was described as a driving factor in choosing DSB over SGB.

From a neuroanatomical point of view, the superior cervical ganglion at C4 and the stellate ganglion at C6 share efferent sympathetic fibers from the T2-T4 thoracic ganglia; however, the route that these fibers take to the brain is different (39). C6 fibers follow the vertebral artery, whereas C4 fibers follow the internal carotid artery (40). Because these arteries supply different regions of the brain, DSBs would increase the intensity of the effect and thus reduce symptoms synergistically (41).

### 3.7 Unilateral vs. bilateral DSB in PTSD

Again, only a single study reported on the differences in the effectiveness of unilateral vs. bilateral DSB in PTSD patients using the Generalized Anxiety Disorder (GAD-7) questionnaire (22). One hundred and sixty-one patients received bilateral DSB while 104 patients received unilateral DSB (unspecified side) in the study. The authors noted that patients receiving bilateral DSB had a score reduction of 9.9 points after 1 week compared with baseline. In comparison, patients with unilateral DSB had a score reduction of 7.5 points after 1 week compared with baseline. This difference was reported to be statistically significant (*p* < 0.001), a trend that was also observed at the one-month follow-up timepoint. At the end of 1 month post DSB, only 67% of the patients with unilateral block demonstrated clinically meaningful improvements in comparison to 81% of the patients with bilateral block (22).

### 3.8 Technique of SGB and DSB

In 17 studies, the anesthetic agent was administered incrementally at the level of 6th cervical vertebra under ultrasound or fluoroscopic guidance. Local anesthesia using 1 mL 1% lidocaine was given optionally to patients based on need. However, there was heterogeneity in the needle size used – 20G Tuohy needle, 22G Quincke needle, and 25G Quincke needle. In all of these studies, either the anterior paratracheal approach or the lateral approach was employed for accessing the targeted area.

For DSB, 1 to 1.5 mL 1% lidocaine was used in all cases and was not offered electively. Only the 22G Quincke needle was used in all patients undergoing DSB. Additionally, the amount of anesthetic agent (bupivacaine) administered in DSB varied from 2 to 7.5 mL with 3 mL being the most common. In cases of bilateral DSB, the DSB on the contralateral side was administered at least 12 to 24 h after the initial DSB. In some cases, the patients were administered the contralateral DSB a week after the initial procedure. This was done to avoid serious airway compromise and vocal cord paresis due to bilateral blocking of the recurrent laryngeal nerve (22). Furthermore, the right DSB was generally preferred to be performed first followed by the left DSB.

### 3.9 Number of SGB/DSB sessions

42% of the patients received a single session of SGB injections across the reviewed studies, 11% of the patients received two sessions of SGB while a single patient received three sessions (31). Most of the patients who received the second or third session reported relapse of the symptoms including nightmares and diminished appetite. The time to relapse from the primary session was 2–6 weeks, with 4 weeks being most frequently reported. However, in one patient, the symptoms reappeared 7 months after the initial session (25). In one of the case series, two out of nine patients were administered a second SGB 4–6 weeks post-primary session (20).

In the case report by Lipov et al. (28) the patient was administered SGB followed by pulsed radiofrequency (PRF) a month later due to symptom relapse. The patient complained of second relapse episode 4 months post-PRF and was administered a second PRF (31). Reportedly, the symptoms persisted and 2 weeks later a second SGB was administered (about 5–6 months post-initial SGB). However, based on the findings from the randomized trial by Rae Olmsted et al. (37) for optimal symptom control, a second session 2 weeks after the primary session has been recommended. The benefits of a second session are also supported by the experience of patients, who report more pronounced and stable effects (29).

Among the patients who received DSB, patients in four studies received DSB once while in two studies DSB was administered twice to the patients. In these two patients who received two DSB sessions, one patient received second DSB supplemented with Ketamine infusions (30) while the second patient received second DSB supplemented with Botox (27). One patient underwent four sessions of DSB with the last three sessions being supplemented with PRF (26).

### 3.10 Follow-up and adverse events

The frequency and duration of the follow-up time-points varied drastically between the studies, attributable to the heterogeneity in the study design and aims. The minimum follow-up time-point was 1 week after the initial SGB/DSB session while the maximum follow-up time-point was 7 months. Twelve studies involving 380 patients (29%) reported no side-effects. Most of the reported adverse events were mild to moderate in nature and resolved spontaneously within 24 h without the need for any additional medical intervention. Pain and stiffness at the injection site in the neck, headache, transient Horner’s syndrome, cough, visual disturbances, temporary hoarseness, difficulty swallowing, hypertension, and bradycardia were the side-effects reported by the patients (37). In a few patients with history of migraines, SGB led to a self-limiting exacerbation episode of migraine. In all other studies, the adverse effects were not reported (likely measured but not mentioned in the text).

## 4 Discussion

Over the decades, SGB has remained a relatively experimental treatment modality, despite the first documented attempts to use it for psychiatric disorders, such as depression, in the late 1940s (42). Only 50 years later in the 1990s, it was found that SGB could be used as a treatment option for PTSD when the procedure was administered for pain management but was found to secondarily ameliorate PTSD symptoms (43). To date, literature remains limited in demonstrating its effectiveness and safety. This is underscored by the fact that SGB is not being offered as a standard of care, at least for PTSD. In addition to PTSD, SGB is used to treat a variety of pain syndromes, including complex regional pain syndrome of the head and upper body (44), peripheral vascular disease (45), post-herpetic neuralgia (46), and postoperative pain (47). A few reports have demonstrated the use of SGB in patients with refractory ventricular arrhythmias (48), Raynaud’s disease (49), and amblyopia caused by quinine toxicity (50).

In the treatment of PTSD, the effectiveness of the technique remains a pivotal unanswered question. In a recent systematic review of the effects of SGB on all psychiatric disorders, the authors suggested that, based on the available evidence, SGB could be a safe and feasible alternative for the treatment of PTSD (10). However, we remain extremely cautious about reaching such a conclusion. Firstly, it is not possible to distinguish between the effects of SGB with adjunctive therapy and SGB alone (38). Secondly, majority of the reporting of clinical data has been done by only two research teams (Lipov et al. and Mulvaney et al.) which makes it difficult to generalize the findings outside their institutions and teams. The lack of blind, unbiased trial data is also a concern. Finally, without a common comparator, such as placebo or usual care/treatment, it is difficult to draw conclusions about the effectiveness of a therapy.

With regard to the choice of anesthetic agent, ropivacaine is emerging as the anesthetic of choice, especially in view of its similar pharmacokinetics to those of bupivacaine. The former also has the advantage of selective nerve blockade (51). Ropivacaine, which is less lipophilic, preferentially targets the thinner A, β, and C pain transmission fibers, resulting in fewer systemic side effects such as hypotension, bradycardia, and motor paralysis (52). However, concerns remain regarding their differing effectiveness. One study compared three doses of spinal intrathecal injections at the lumbar L2-L3 level (4 mg, 6 mg, and 8 mg) of 0.25% bupivacaine and 0.25% ropivacaine in healthy volunteers (53). Bupivacaine was reported to be nearly twice as potent as ropivacaine (2:1) and equipotent doses had similar recovery times. The authors also noted that ropivacaine was more likely to cause injection site pain (53). However, a systematic review suggested that when used at the ideal concentrations of 0.50–0.75%, the choice of anesthetic between ropivacaine, levobupivacaine, and racemic bupivacaine does not appear to affect clinical success (54).

The use of ultrasound and fluoroscopic techniques has not only reduced the rate of iatrogenic complications but also significantly reduced the concentration of anesthetic required to achieve SGB. Several studies have shown that an optimal concentration of 5 mL of 0.25% bupivacaine is sufficient to achieve pain control, and doses in excess of 20 mL may result in the uncontrolled spread of the agent to other regions (55, 56). The addition of adjuvants such as clonidine (α₂-adrenergic agonist) and methylprednisolone (steroid) to anesthetic injections has also been suggested. Clonidine prolongs the motor block and the duration of anesthesia—approximately 1 h and 40 min with ropivacaine and nearly 4 h with bupivacaine (57). The addition of steroids to anesthesia remains controversial. There is limited evidence to support their beneficial role in suppressing the anti-inflammatory response and prolonging the anesthetic effect (58). Furthermore, in SGB used to treat complex regional pain syndrome, adding clonidine or methylprednisolone to ropivacaine did not result in significant differences in pain, edema, or overall patient satisfaction (59).

Laterality has also become debatable in recent years, with recent evidence suggesting a much more complex neural control than previously thought. Traditionally, the right-sided block has been favored because of the hemispheric asymmetry theory of autonomic arousal—right hemisphere modulates sympathetic, left hemisphere modulates parasympathetic responses (60). Indeed, electroencephalographic studies have shown that PTSD patients tend to have asymmetrical right-temporal lobe activation (61), a phenomenon also observed when PTSD patients are exposed to traumatic material (62). However, a bi-hemispheric autonomic model of autonomic control has been postulated based on the demonstration that patients with PTSD can be grouped based on temporal asymmetry into right-dominant, left-dominant, and symmetrical groups (60, 63). This heterogeneity has been called “*functional neuroanatomical* *variability*” by Mulvaney et al. (34). This rationale also led the authors to conclude that in patients with treatment-resistant right-sided SGB, a left-sided SGB may show significant improvement in clinical symptomatology (34). What factors might influence such differences in autonomic laterality physiology—sex, age, type of PTSD trauma, symptom severity—cannot be determined based on available literature. Further studies will be necessary for a more complete understanding of this issue.

Interestingly, in a study by (14) it was demonstrated that factors such as age, history of suicide attempts, or use of medications does not significantly affect the PCL scores in both male and female PTSD patients post-SGB. However, the authors showed that the reduction in PCL scores was significantly different based on military or non-military background of the participants in both male (mean difference 6.94; *p* = 0.012) and female (mean difference 10.92; *p* = 0.020) participants. These findings are crucial since a key drawback limiting the generalizability of SGB use has been the overrepresentation of military personnel and veterans in the studies testing the effectiveness of SGB. In the same study, the authors also reported that the type of trauma exposure was not a confounding factor when considering the effectiveness of SGB in PTSD patients. All trauma types demonstrated statistically significant reduction in PCL scores post-SGB for both male and female participants except for bullying/hazing, parental issues, domestic violence, and injury/death of a loved one. The non-significant findings in these subgroups could be explained by the small (<5) sample sizes. Nonetheless, the finding that military personnel showed more sensitivity to SGB than civilians was hypothesized by the authors to be a result of higher baseline PCL score in military personnel and sensitivity differences between the PCL-M and PCL-C questionnaires to detect the severity of PTSD (14).

In terms of safety, it has been reported that the rate of severe complications following SGB is around 1.7 complications per 1,000 blocks (64). Although this makes the procedure relatively safe, especially when performed under ultrasound or fluoroscopic guidance, it must be kept in mind that SGB remains an invasive procedure and may lead to local and systemic complications. Localized risk of injury to anatomically adjacent muscles and vessels remains. There is a risk of intravascular infection and injection, which in turn can lead to convulsions, hematomas, seizures, and even local anesthetic systemic toxicity (65). Systemically, the most common complications are hoarseness and dizziness, followed by a short-lasting cough, probably from recurrent laryngeal paralysis (66).

Despite the current literature supporting potential benefits of SGB for PTSD, more supporting evidence is needed before the procedure can be mainstreamed as an alternative to current recommendations. Furthermore, the implementation and adoption of the procedure outside of the US remains to be seen. The physician’s skill and expertise in administering SGB also plays a limiting role in its wider validation. It is pertinent to note that SGB and anesthetics ropivacaine and bupivacaine are Food and Drug Administration (FDA) approved for surgery and pain management, though their use in PTSD is considered “off-label” according to both FDA and US Department of Defense management guidelines (67). This could be one of the reasons eclipsing the lack of wider adoption and literature. However, the results from more and more studies in the coming years could open the door for FDA approval of ropivacaine and bupivacaine for SGB in the treatment of PTSD, especially if there was more confidence in it and more practitioners were trained to administer it.

In fact, a post-procedure qualitative review of 110 patients who underwent SGB found that 100% of the participants were willing to have a repeated SGB session, with 95% willing to take as many sessions as it takes (68). Furthermore, all participants agreed to recommend the procedure to their family and friends. 50% of the respondents said that the side effects after SGB were less in comparison with other treatment modalities they had tried with 89% of the respondents reporting no to mild discomfort post-procedure (68). Similar results were reported by a cohort of 53 military and civilian psychiatrists, social care workers, and nurses who deal with PTSD patients. 65% of the professionals reported SGB to be very beneficial for the patient with no professional considering the procedure as harmful or not helpful (69). Additionally, the professionals considered SGB at par with all eight treatment modalities listed in the 2017 American Psychological Association (APA) Clinical Practice Guideline (CPG) for PTSD (69).

Results from these two studies indicate a growing acknowledgement and desire for adoption of SGB as a mainstream treatment modality in the treatment of PTSD. Accordingly, the technique could potentially be a viable alternative in treatment-resistant or refractory cases of PTSD. Such newer avenues are needed since remission of PTSD has been heterogeneous even for strongly recommended first line treatments such as CBT, EMDR, and prolonged exposure therapy (PE) (6). For example, in PE, 41–95% of participants did not meet PTSD diagnostic criteria after treatment, while in cognitive processing therapy (CPT) the rate was 30–97% after treatment, and in CBT it was 61–82.4% after treatment (70). While adjunctive pharmacotherapy may provide symptomatic relief, it does not target the core pathogenetic processes, as was also reported in the studies included in our review.

## 5 Limitations

Heterogeneity in the design of the trials, lack of standardized placebo-controlled trials, predominantly male participants, and small sample size are the main limitations in terms of study designs included in the present review. Furthermore, one must keep in mind that the generalizability of the results of any study or report is directly influenced by the highly individualized responses that are part of the SGB’s effectiveness evaluation (visual analog scale, self-reporting questionnaires). The use of concomitant therapy (pharmacological and non-pharmacological) and different numbers of SGB sessions were other limiting factors. Additionally, the presence of comorbid conditions like insomnia and nightmares are associated with poorer PTSD treatment outcomes. In a recent randomized controlled trial, it was demonstrated that CPT for PTSD followed by CBT for insomnia and nightmares was the most effective in improving symptoms in comparison with CPT for PTSD alone (71).

Finally, it is not possible to accurately estimate the number of participants who actually lost their PTSD diagnosis or did not fulfill PTSD criteria after SGB/DSB. This is mainly due to two factors – first, the limited follow-up duration reported in the studies and second, the effects of SGB seem to wear off after some time, leading to an increase in PCL-5 scores on reassessment. Hence, many patients required additional sessions. The utility of bilateral SGB or DSB also needs further investigations due to limited comparative studies. However, bilateral administration of block should be done carefully due to its ill-effects on the baroreflex sensitivity and cardiac vagal modulation (72). To overcome these challenges, newer techniques are being reported as case reports such as the use of hydro-dissection of the cervical plexus using 5% dextrose (73). These techniques would also need further investigations and trials to confirm their clinical effectiveness.

## 6 Future double-blind clinical trials

It is clear that more evidence is needed, especially that is derived from well-designed randomized clinical trials. However, one of the most important drawbacks noted in clinical trials is the issue of blinding participants and physicians. Transient ipsilateral Horner’s syndrome is commonly seen after successful SGB which risks unblinding of the participants. To avoid this, Rae Olmsted et al. (37) informed patients in both sham and SGB group about Horner’s syndrome, though acknowledged that complete unbiased blinding was not possible to achieve.

To overcome this limitation, it has been suggested to use PRF which achieves SGB without causing Horner’s syndrome (74, 75). As noted in two studies in our review, PRF has secondary benefits of conferring longer lasting treatment effects in comparison with anesthetic mediated SGB (26, 31). PRF is believed to selectively deactivate nerve cell membranes of the small-diameter nerve fibers due to membrane depolarization caused by the electromagnetic field (76). Alternatively, it has also been suggested to keep the trial participants in the observation room for enough time (2–48 h) for the signs of Horner’s syndrome to resolve (75). Regarding blinding of the physicians, it has been advised to have two teams—a physician team and a research team with no interactions between them (75).

Finally, it is necessary to separate the clinical effects attributable to the effects of SGB and to those of concomitant pharmacotherapy. As seen from the studies included in our review, several patients were reported to take antihypertensives like clonidine and beta-blockers, both of which are known to inhibit sympathetic activity by either peripheral blockade of adrenergic receptors or by central mechanisms. Additionally, many psychotropic medications (antidepressants and antipsychotics) have indirect effects on sympathetic tone and may present a confounding factor when measuring the effectiveness of SGB.

## 7 Conclusion

Current literature remains limited regarding the potential effectiveness and safety profile of SGB, leading to its exclusion as a standard treatment option for PTSD. While preliminary studies and case reports provide generally supporting evidence of SGB as a safe alternative for PTSD, caution is warranted due to inconsistent results and the lack of blinded, unbiased trial data. Further studies are needed to evaluate the effectiveness of the block in terms of anesthetic dosage, frequency, and use of concomitants. In addition, laterality, particularly the preference for right-sided block, has been challenged by evidence suggesting a bi-hemispheric model of autonomic control that needs to be clinically validated. Careful and extensive clinical research studies are needed to address the above raised pertinent questions and issues before the introduction of the technique as a mainstream adjunct modality.

## Data availability statement

The original contributions presented in the study are included in the article/Supplementary material, further inquiries can be directed to the corresponding author.

## Author contributions

SP: Conceptualization, Formal analysis, Investigation, Methodology, Project administration, Validation, Writing – original draft, Writing – review & editing. NJ: Conceptualization, Data curation, Formal analysis, Funding acquisition, Investigation, Methodology, Project administration, Resources, Software, Validation, Visualization, Writing – original draft, Writing – review & editing. TU: Data curation, Investigation, Writing – original draft. IR: Data curation, Investigation, Writing – original draft. SKh: Data curation, Investigation, Writing – original draft. VS: Data curation, Investigation, Writing – original draft. SKo: Data curation, Investigation, Writing – original draft. MH: Data curation, Investigation, Writing – original draft. NK: Data curation, Investigation, Writing – original draft. AR: Data curation, Methodology, Writing – original draft. LR: Project administration, Resources, Supervision, Validation, Writing – review & editing. AB: Project administration, Resources, Supervision, Validation, Writing – review & editing.
