# Supplementary material for: Sympathetic nerve blocks for posttraumatic stress disorder: an evidentiary review for future clinical trials
Source: Front Psychiatry. 2023 Dec 22;14:1309986. doi: 10.3389/fpsyt.2023.1309986 (PMC10771322; doi:10.3389/fpsyt.2023.1309986)
Supplement: Supplementary file 1 [file Data_Sheet_1.pdf]

SUPPLEMENTARY MATERIAL

Frontiers in Psychiatry

Sympathetic Nerve Blocks for Posttraumatic Stress Disorder: An Evidentiary Review for Future Clinical Trials

Sakshi Prasad*, Nityanand Jain*, Tungki Pratama Umar, Igor Radenkov, Sirwan Khalid Ahmed, Virginia Sakagianni, Sofia Kollia, Mohmed Junaid Hingora, Nikita Kumari, Amir Reza Akbari, Lubova Renemane, and Anil Bachu

DOI: 10.3389/fpsyt.2023.1309986

***Corresponding Authors**

Table 1.
Risk of Bias (RoB) Assessment for Case Reports

Checklist used: JBI Critical Appraisal Tool for Case Reports

Criteria	Block et al. (2023)	Kuo & Nicklay (2023)	Lipov et al. (2008)	Lipov et al. (2010)	Lipov et al. (2013)	Lipov et al. (2023)
Were patients' demographic characteristics clearly described?	Yes	Yes	Yes	Yes	Yes	Yes
Was the patient's history clearly described and presented as a timeline?	Yes	Yes	Yes	Yes	Yes	Yes
Was the current clinical condition of the patient on presentation clearly described?	Yes	Yes	Yes	Yes	Yes	Yes
Were diagnostic tests or assessment methods and the results clearly described?	Yes	Yes	No	No	Yes	Yes
Was the intervention(s) or treatment procedure(s) clearly described?	Yes	Yes	Yes	Yes	Yes	Yes
Was the post-intervention clinical condition clearly described?	Yes	Yes	Yes	Yes	Yes	Yes
Were adverse events (harms) or unanticipated events identified and described?	Yes	Yes	No	No	No	No
Does the case report provide takeaway lessons?	Yes	Yes	Yes	Yes	Yes	Yes
**Overall Perceived risk**	**Low**	**Low**	**Moderate**	**Moderate**	**Low**	**Low**

Table 2.
Risk of Bias (RoB) Assessment for Case Series

Checklist used: JBI Critical Appraisal Tool for Case Series

Criteria	Aliño et al. (2013)	Hicky et al., 2012	Lipov & Faber, 2023	Lynch et al., 2023	Mulvaney et al., 2010	Mulvaney et al., 2014	Mulvaney et al., 2015
Were there clear criteria for inclusion in the case series?	Yes	Yes	No	Yes	Yes	Yes	Yes
Was the condition measured in a standard, reliable way for all participants included in the case series?	Yes	Yes	Yes	Yes	Yes	Yes	Yes
Were valid methods used for identification of the condition for all participants included in the case series?	Yes	Yes	Yes	Yes	Yes	Yes	Yes
Did the case series have consecutive inclusion of participants?	N/A	Yes	No	Yes	No	Yes	Yes
Did the case series have complete inclusion of participants?	N/A	Yes	No	Yes	No	Yes	Yes
Was there clear reporting of the demographics of the participants in the study?	Yes	Yes	Yes	Yes	Yes	Yes	Yes
Was there clear reporting of clinical information of the participants?	Yes	Yes	Yes	Yes	Yes	Yes	Yes
Were the outcomes or follow up results of cases clearly reported?	Yes	Yes	Yes	Yes	Yes	Yes	Yes
Was there clear reporting of the presenting site(s)/clinic(s) demographic information?	Yes	Yes	Yes	Yes	No	Yes	Yes
Was statistical analysis appropriate?	N/A	N/A	N/A	Yes	No	Yes	Yes
**Overall Perceived risk**	**Low**	**Low**	**Moderate**	**Low**	**Moderate**	**Low**	**Low**

Table 3.
Risk of Bias (RoB) Assessment for Retrospective Cohort Studies

Checklist used: JBI Critical Appraisal Tool for Cohort Studies

Criteria	Lipov et al. (2012)	Lipov et al. (2022)	Lynch et al. (2016)	Mulvaney et al. (2020)	Mulvaney et al. (2021)	Odosso & Petta (2021)
Were the two groups similar and recruited from the same population?	Yes	Yes	Yes	Yes	Yes	Yes
Were the exposures measured similarly to assign people to both exposed and unexposed groups?	Yes	Yes	Yes	Yes	Yes	Yes
Was the exposure measured in a valid and reliable way?	Yes	Yes	Yes	Yes	Yes	Yes
Were confounding factors identified?	No	Yes	Yes	No	Yes	Yes
Were strategies to deal with confounding factors stated?	No	Yes	Yes	No	Yes	Yes
Were the groups/participants free of the outcome at the start of the study (or at the moment of exposure)?	No	No	Yes	No	No	Yes
Were the outcomes measured in a valid and reliable way?	Yes	Yes	Yes	Yes	Yes	Yes
Was the follow up time reported and sufficient to be long enough for outcomes to occur?	Yes	Yes	Yes	Yes	Yes	Yes
Was follow up complete, and if not, were the reasons to loss to follow up described and explored?	No	Yes	Yes	No	Yes	Yes
Were strategies to address incomplete follow up utilized?	No	No	Yes	No	No	No
Was appropriate statistical analysis used?	Yes	Yes	Yes	Yes	Yes	Yes
**Overall Perceived risk**	**Moderate**	**Low**	**Low**	**Moderate**	**Moderate**	**Low**

Table 4.
Risk of Bias (RoB) Assessment for Retrospective Randomized Controlled Trials

Checklist used: ROB-2

Criteria	Hanling et al. (2016)	Rae Olmsted et al. (2020)
Risk of bias arising from the randomization process	Moderate	Low
Risk of bias due to deviations from the intended interventions (effect of assignment and adhering to intervention)	Low	Low
Missing outcome data	Low	Low
Risk of bias in measurement of the outcome	Low	Low
Risk of bias in selection of the reported result	Low	Low
**Overall Perceived risk**	**Moderate**	**Low**

Table 5.
Risk of Bias (RoB) Assessment for Retrospective Non-Randomized Controlled Trials

Checklist used: ROBINS-I

Criteria	Peterson et al. (2022)
Bias due to confounding	Low
Bias in selection of participants in the study	Moderate
Bias in classification of interventions	Low
Bias due to deviations from intended interventions	Low
Bias due to missing data	Low
Bias in measurement of outcomes	Low
Bias in selection of the reported results	Low
**Overall Perceived risk**	**Low**
